# Canine models of human amelogenesis imperfecta: identification of novel recessive *ENAM* and *ACP4* variants

**DOI:** 10.1007/s00439-019-01997-8

**Published:** 2019-03-15

**Authors:** Marjo K. Hytönen, Meharji Arumilli, Eva Sarkiala, Pekka Nieminen, Hannes Lohi

**Affiliations:** 1 Department of Veterinary Biosciences, University of Helsinki, Helsinki, Finland; 2 Research Programs Unit, Molecular Neurology, University of Helsinki, Helsinki, Finland; 3 Folkhälsan Research Center, Helsinki, Finland; 4 Department of Equine and Small Animal Medicine, University of Helsinki, Helsinki, Finland; 5 Evidensia Tammisto, Vantaa, Finland; 6 Orthodontics, Oral and Maxillofacial Diseases, University of Helsinki, Helsinki, Finland

## Abstract

Amelogenesis imperfecta (AI) refers to a genetically and clinically heterogeneous group of inherited disorders affecting the structure, composition, and quantity of tooth enamel. Both non-syndromic and syndromic forms of AI have been described and several genes affecting various aspects of the enamel physiology have been reported. Genetically modified murine models of various genes have provided insights into the complex regulation of proper amelogenesis. Non-syndromic AI occurs spontaneously also in dogs with known recessive variants in *ENAM* and *SLC24A4* genes. Unlike rodents with a reduced dentition and continuously erupting incisors, canine models are valuable for human AI due to similarity in the dental anatomy including deciduous and permanent teeth. We have performed a series of clinical and genetic analyses to investigate AI in several breeds of dogs and describe here two novel recessive variants in the *ENAM* and *ACP4* genes. A fully segregating missense variant (c.716C>T) in exon 8 of *ENAM* substitutes a well-conserved proline to leucine, p.(Pro239Leu), resulting in a clinical hypomineralization of teeth. A 1-bp insertion in *ACP4* (c.1189dupG) is predicted to lead to a frameshift, p.(Ala397Glyfs), resulting in an abnormal C-terminal part of the protein, and hypoplastic AI. The *ENAM* variant was specific for Parson Russell Terriers with a carrier frequency of 9%. The *ACP4* variant was found in two breeds, Akita and American Akita with a carrier frequency of 22%. These genetic findings establish novel canine models of human AI with a particular interest in the case of the ACP4-deficient model, since ACP4 physiology is poorly characterized in human AI. The affected dogs could also serve as preclinical models for novel treatments while the breeds would benefit from genetic tests devised here for veterinary diagnostics and breeding programs.

**Electronic supplementary material:**

The online version of this article (10.1007/s00439-019-01997-8) contains supplementary material, which is available to authorized users.

## Introduction

Enamel is a mineralized tissue largely composed of hydroxyapatite crystals and covers the dental crown with uniquely hard properties offering insulation and resistance to fractures and wear (Witkop and Sauk 1976). Enamel is generated by ameloblasts in a coordinated process including presecretory, secretory, transition, and maturation stages (Smith 1998; Lacruz et al. 2012). Deviations from normal genetic programming during development may result in amelogenesis imperfecta (AI). AI refers to a genetically and clinically heterogeneous group of genetic conditions affecting the structure, composition and quantity of tooth enamel, and can be classified into hypoplastic, hypomaturation and hypomineralized types (Witkop 1988; Aldred et al. 2003). Hypoplastic AI refers to the altered thickness of the enamel and can be classified to various (pitted, local, smooth and rough) subtypes. The hypomaturation and hypomineralization types do not affect the enamel thickness, but affects enamel matrix composition and contribute to softer abrasion-sensitive enamel, respectively (Witkop 1988).

The reported prevalence of human AI varies from 1 in 14,000 to as high as 1 in 700 (Backman and Holm 1986; Smith et al. 2017a). Implications of AI to patients vary from eating difficulties to negative social outcomes and poor aesthetics (Hashem et al. 2013). AI cannot be cured and interventions focus on aesthetics and maintaining occlusal height, tooth function, and natural dentition (Dashash et al. 2013). A recent review lists over 90 non-syndromic or syndromic conditions with an enamel phenotype, including at least 71 with a known molecular etiology or loci (Wright et al. 2015). Non-syndromic AI shows genetic heterogeneity with X-linked, autosomal dominant or autosomal recessive inheritance patterns (Lench and Winter 1995; Dong et al. 2000; Hart et al. 2003). Disease-causing variants have been identified in 18 genes, from which the variants in four genes account for the majority of the AI cases: family with sequence similarity 83 member H (*FAM83H*; MIM *611927; Kim et al. 2008), family with sequence similarity 20 member A (*FAM20A*; MIM *611062; O’Sullivan et al. 2011), enamelin (*ENAM*; MIM *606585; Rajpar et al. 2001; Mardh et al. 2002) and amelogenin (*AMELX*; MIM*300391; Kim et al. 2004). A recent online resource (http://dna2.leeds.ac.uk/LOVD/) details ~ 200 published AI-causing mutations in all 18 known genes. Gene discoveries are helpful to distinguish AI from other frequent enamel defects such as fluorosis and molar incisor hypomineralization as well as serious systemic illness (Salanitri and Seow 2013).

Phenotyping teeth from AI patients is not straightforward due to age-related post-eruptive changes and unavailability of embryonic teeth, which precludes the study of amelogenesis itself. Therefore, mouse models have been highly important to AI research. Several transgenic mouse lines have been generated and the list of available models has been recently summarized (Smith et al. 2017a). Amelogenin-null mice demonstrate the importance of amelogenin in the regulation of enamel thickness and in the organization of crystal patterns (Gibson et al. 2001). Enamelysin (Mmp20)-deficient mice fail to process amelogenin resulting in serious enamel defects (Caterina et al. 2002). Mice overexpressing *Mmp20* exhibit significant reductions in enamel thickness, volume, and mineral density in their molars (Shin et al. 2018). Enamelin-deficient mice present with defects in enamel mineralization (Hu et al. 2008), while an enamelin overexpression model suggests that an adequate quantity of enamelin is essential for normal enamel formation (Hu et al. 2014).

Spontaneous forms of AI have been reported also in dogs. Particular interest in the canine dental models is due to the fact that unlike rodents, dogs have both deciduous and permanent teeth similar to humans. A 5-bp deletion in exon 10 of *ENAM* was reported in Italian Greyhounds (Gandolfi et al. 2013) and a 21-bp duplication in exon 17 of *SLC24A4* in Samoyeds (Pedersen et al. 2017). We have characterized other AI-affected dog breeds and describe here the clinical and genetic characteristics of two new models with recessive variants in *ENAM* and acid phosphatase 4 (*ACP4*). This is particularly important in the case of *ACP4*, where the association with AI has been quite recently demonstrated and no mouse model exists yet. We describe here the first spontaneous animal model for *ACP4*.

## Materials and methods

### Study cohort and DNA extraction

EDTA-blood samples were collected for DNA isolation from 372 Parson Russell Terriers (PRTs) and 159 Akitas. For additional variant screening we used samples from our biobank as follows: 97 Jack Russell Terriers, 197 American Akitas, 36 Alaskan Malamutes, 9 Kais and 3 Hokkaidos. Genomic DNA was extracted from white blood cells using a semi-automated Chemagen extraction robot (PerkinElmer Chemagen Technologie GmbH, Germany), and concentration was measured using a Qubit fluorometer (Thermo Fisher Scientific, USA) or Nanodrop ND-1000 UV/Vis spectrophotometer (Nanodrop technologies, USA). The samples were stored at − 20 °C. Sample collection was ethically approved by the Animal Ethics Committee of State Provincial Office of Southern Finland (ESAVI/343/04.10.07/2016).

### Clinical and phenotypic examinations

Dental and oral examinations were performed on every dog and full mouth dental radiographs were taken by a board-certified veterinary dentist. Blood samples of affected PRTs were taken for complete blood count (CBC) and serum biochemistry, and EDTA-blood samples were obtained for DNA extraction.

Microcomputed tomography (microCT) of affected and unaffected Akita deciduous premolars was performed with a SkyScan 1272 instrument (Bruker microCT, Kontich, Belgium) with a voxel size of 12 µm. Images were processed and analyzed with Image J software (Wayne Rasband, National Institute of Health, imagej/nih/gov/ij).

### Genetic analyses

Genome-wide SNP genotyping of five affected and eight unaffected Akitas was performed using Illumina’s canine HD 173K SNP array at Neogen Genomics (Lincoln, NE, USA). Quality control was carried out using a SNP genotyping call rate of > 95%, minor allele frequency of > 0.05 and an array call rate > 95%. After frequency and genotype pruning, 64,314 SNPs remained for analysis and five cases were kept for further analysis. Quality control and homozygosity mapping was carried out using PLINK 1.07 (Purcell et al. 2007). Homozygosity mapping was performed by retrieving pools of overlapping runs of homozygosity with SNP density set to one SNP per 200 kb. The genotype data are available for further use upon request.

Whole-exome sequencing (WES) of one affected Parson Russell Terrier and one Akita were prepared using the libraries of Roche NimbleGen SeqCap EZ target enrichment design (exome-1.0) (Broeckx et al. 2014). The samples were sequenced with a read length of 150 bp (paired-end reads, 2 × 75 bp) yielding ~ 190X mean coverage. The libraries for another affected Akita was prepared using the Roche NimbleGen SeqCap EZ target enrichment design 140702_canFam3_exomeplus_BB_EZ_HX1 with a capture size of 152 Mb (Broeckx et al. 2015) and was sequenced with a read length of 300 bp (paired-end reads, 2 × 150 bp). The library preparation and sequencing were performed for all the samples at the Biomedicum Functional Genomics Unit (University of Helsinki, Finland) with the Illumina NextSeq500 platform. The reads were mapped using the Burrows-Wheeler Aligner [BWA, (Li and Durbin 2009)] version 0.7.12-r1039 to the canine reference genome (canFam3.1) and Picard tools (http://broadinstitute.github.io/picard/) was used to sort the mapped reads by coordinates and to mark duplicates. Indel realignment, base-quality score recalibration and variant calling in GVCF mode were performed with the HaplotypeCaller 3.5.0 in Genome Analysis Tool Kit (GATK) (McKenna et al. 2010) and the variants were genotyped using GenotypeGVCFs. The variants were annotated with ANNOVAR (Wang et al. 2010) to Ensembl, RefGene, Broad and FEELnc (Wucher et al. 2017) gene databases. The details of the sequencing, mapping quality and variant data are seen in Table 1. Additionally, we used WGS variant data from publicly available genomes and from our other studies (648 dogs) as controls in variant filtering (Online Resource 1). To identify potential causative variants, we performed variant filtering with our in-house variant database built and queried using Genotype Query Tools (Layer et al. 2016). The exome sequencing data of all affected dogs are available at the NCBI Sequence Read Archive with BioSample accessions SAMN10662431, SAMN10662432, SAMN10662433 and SAMN10662434. The publicly available control variant data can be found under project accession codes PRJEB10823, PRJEB13139, PRJEB13468, PRJEB13723, PRJEB14110, PRJEB14840, PRJEB16012, PRJEB4544, PRJEB5500, PRJEB5874, PRJEB5875, PRJEB6076, PRJEB6079, PRJEB7734, PRJEB7735, PRJEB7736, PRJEB7903, PRJEB9437, PRJEB9590, PRJEB9591, PRJNA266585, PRJNA319610, PRJNA357866, PRJNA360671, PRJNA380744, PRJNA394814, SRP126148.

**Table 1:** The details of the whole-exome sequencing of the three affected dogs used in the study

Sample	Exome	Total reads	% QC mapped reads (%)	Mean coverage	Homozygous variants	Total variants
Akita 1	Exome-1.0	163,242,256	100	195 ×	53,033	99,929
Akita 2	Exome plus	68,529,557	99.6	49 ×	165,587	292,287
PRT	Exome-1.0	129,251,750	100	191 ×	35,408	88,293

Prediction of the variant pathogenicity was assessed using PROVEAN (Choi et al. 2012; Choi and Chan 2015) and PolyPhen-2 (Adzhubei et al. 2010) programs. NCBI reference sequence XM_539305.4 and XP_539305.3 for ENAM and XM_541473.2 and XP_541473.2 for ACP4 were used to count the nucleotide and amino acid positions.

### Variant screening

The variants of *ENAM* (XM_539305.4:c.716C>T) and *ACP4* (XM_541473.2:c.1189dupG) were genotyped using standard PCR and Sanger sequencing to confirm the association of the variants with AI in each breed. The primer pair used for *ENAM*:c.716C>T was 5′-CAGCACGGTGGGAAACAAAG-3′ and 5′-CACTCTGACTCCCTCCAGGA-3′ and for *ACP4*: c.1189dupG 5′- AAATGGGCGCACACAGTAAG-3′ and 5′-CCCAAGACTCACACTCCCAT-3′. The primers were designed with Primer 3 software (Untergasser et al. 2012; Koressaar and Remm 2007) and the amplified PCR products were sequenced with a capillary sequencer at the Institute for Molecular Medicine Finland core facility (FIMM, Technology Centre, University of Helsinki, Helsinki, Finland). The sequences were analyzed using the Sequencher 5.3 software (GeneCodes, USA).

## Results

### Amelogenesis imperfecta in Parson Russell Terriers

**Fig. 1:**
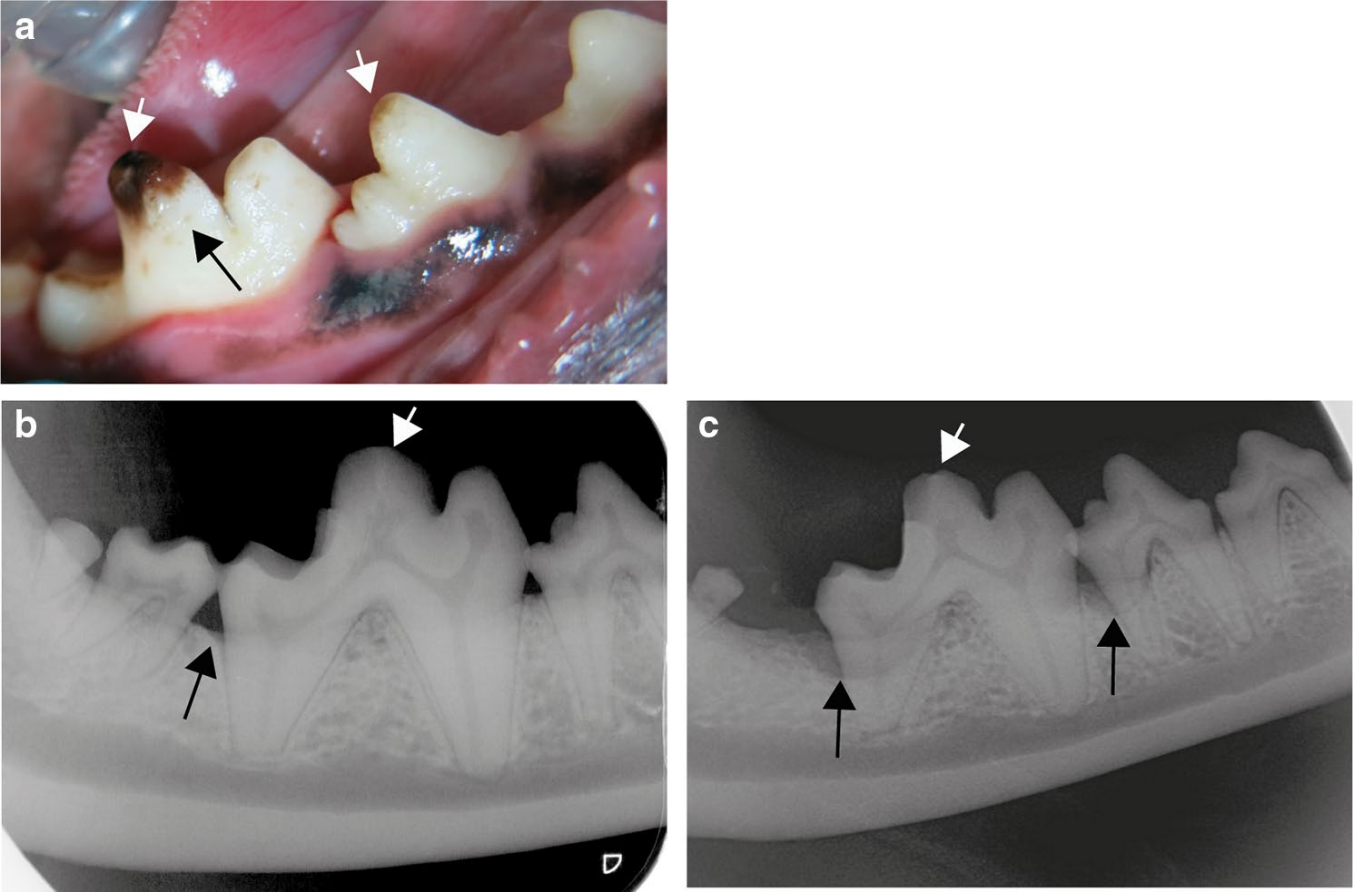
Clinical features of amelogenesis imperfecta in Parson Russell Terriers. **a** A photograph of the right mandibular PM4 and M1 tooth of Dog 3 at 4 years of age after the teeth had been scaled and polished. The enamel is uneven and spotted with tiny pits (black arrow). At the crown tip there is abrasion and staining of the exposed dentin (white arrows). **b** A dental radiograph of the right mandibular PM4, M1 and M2 teeth of Dog 3 at 4 years of age. There is very mild alveolar bone loss distally to M1 (black arrow). The cusp tip of M1 is flattened from abrasion (white arrow). **c** A dental radiograph of the right mandibular PM4 and M1 teeth of Dog 3 at age of 6.5 years. There is moderate alveolar bone loss at the distal root of PM4 and M1 (black arrows). The cusp tip of M1 is flattened from abrasion (long arrow). M2 has been earlier extracted due to periodontitis

Three PRT puppies, two females and a male, were presented for a dental consultation since the owner of each dog had observed that the enamel of the recently erupted permanent teeth appeared dull. There was no history of illness during the life of the dogs and physical examination did not reveal any pathology. The results of CBC and blood chemistry were within normal limits. Dogs were anesthetized for the first dental examination at 6 (dog 2), 8 (dog 1) and 10 (dog 3) months of age. A dental and oral examination was performed and full mouth dental radiographs were taken. There was gingivitis, plaque and a small amount of dental calculus on all dogs’ teeth. Dental examination revealed that all teeth in each dog lacked normal hard and shiny enamel (Fig. 1a). The surface of the crowns appeared dull, rough and hypocalcified. In dental radiographs, enamel was not clearly visible but there was no other pathology (Fig. 1b). After scaling and polishing dentin bonding was performed for all teeth in order to reduce possible sensitivity. There were no deep pits or defects that would have required composite restorations. Dog 1 and 3 returned for dental examinations and teeth cleaning several times almost every year during a follow up period of over 8 years. Both dogs had a moderate amount of plaque and calculus buildup on their teeth annually. Already at early age both Dog 1 and Dog 3 (aged 3 years and 2 years, respectively) had incisor teeth extracted due to advanced periodontitis. Additionally, at the same age teeth displayed mild abrasion of the crown tips. Periodontitis at mandibular M1 teeth of Dog 3 had advanced from mild to moderate from age 4 to 6.5 years of age (Fig. 1b, c). By the age of 8 years, Dog 3 had a total of 20 teeth extracted due to advanced periodontitis. Dog 2 was only seen twice and the last visit was at the age of 2 years.

#### Whole-exome sequencing identifies a missense variant in ENAM

**Fig. 2:**
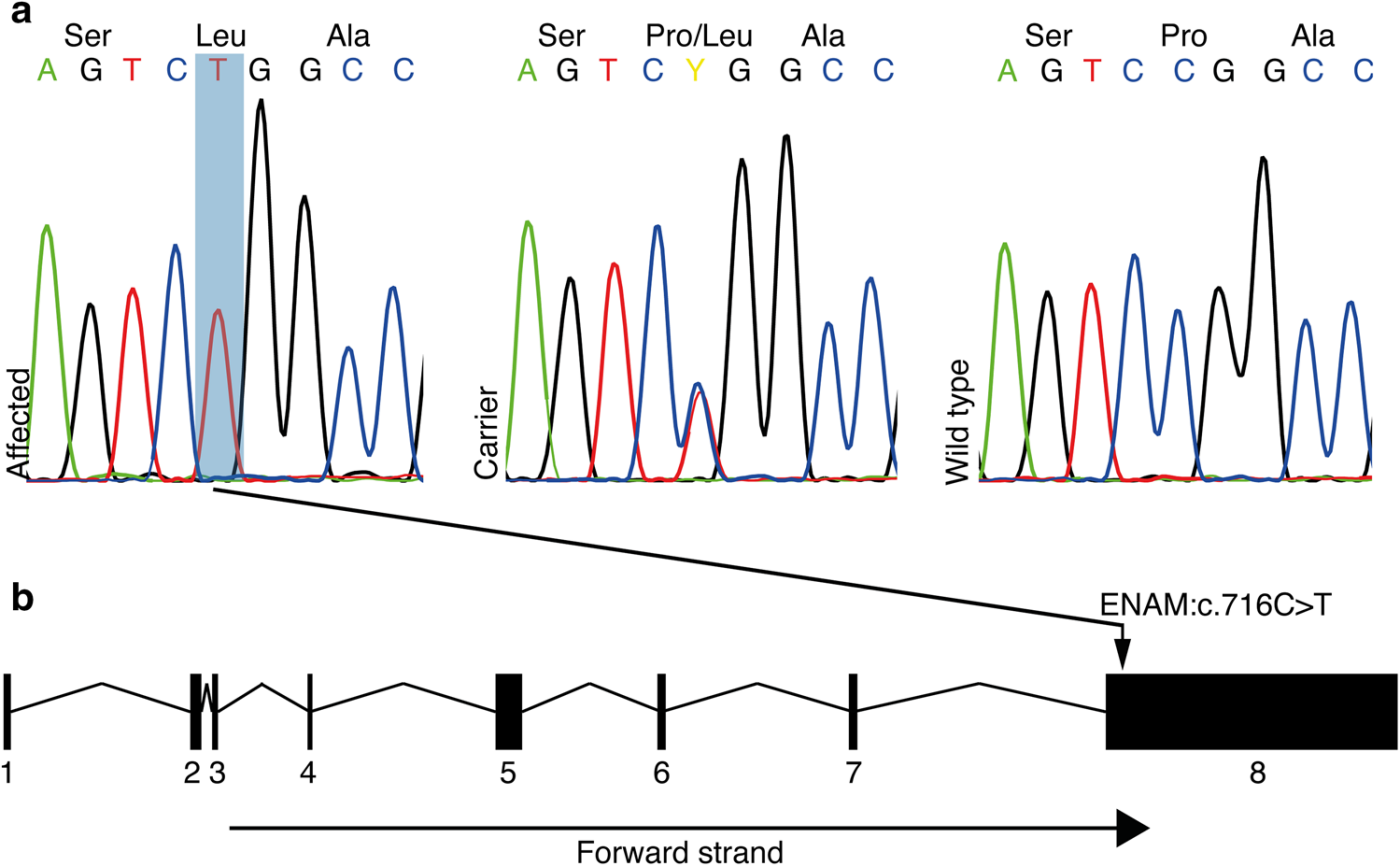
Identification of a missense variant in *ENAM* associated with AI in PRTs. **a** The Sanger sequencing chromatograms showing *ENAM*:c.716C>T variant as homozygous in affected dog and heterozygous and wild-type in carrier and normal, respectively. **b***ENAM* gene contains eight exons and the variant c.716C>T is located in the exon 8

We performed exome sequencing on one case to identify the disease-causing variant. The variant data were filtered against 648 control genomes (Online Resource 1) assuming an autosomal recessive inheritance of the disease. As a result, altogether eight variants with predicted effect on protein remained (Online Resource 2) including a missense variant in exon 8 in enamelin (*ENAM*:c.716C>T) (Fig. 2), an excellent candidate gene because of its known association with AI. *ENAM*:c.716C>T results in a proline to leucine substitution, p.(Pro239Leu), changing an amino acid which is relatively conserved across the phylogenetic tree (Online Resource 3). In silico analysis of the pathogenicity of the variant using PROVEAN Protein and PolyPhen-2 tools both predicted a highly deleterious change with scores − 8.286 and 0.991, respectively.

To evaluate the segregation pattern and carrier frequency, we genotyped the variant by Sanger sequencing in a total cohort of 372 PRTs including the three affected littermates (Fig. 2a). The variant was found to be homozygous in all three affected littermates, heterozygous in 33 dogs and wild-type in 336 dogs, indicating a full segregation with disease in the PRT breed. Carrier frequency in the cohort was 9% (33/372). In summary, the variant showed full segregation with the disease in PRT breed. We also screened the variant in a cohort of 94 Jack Russell Terriers, a closely related breed, but all were homozygous for the wild-type allele.

### Amelogenesis imperfecta in Akitas

**Fig. 3:**
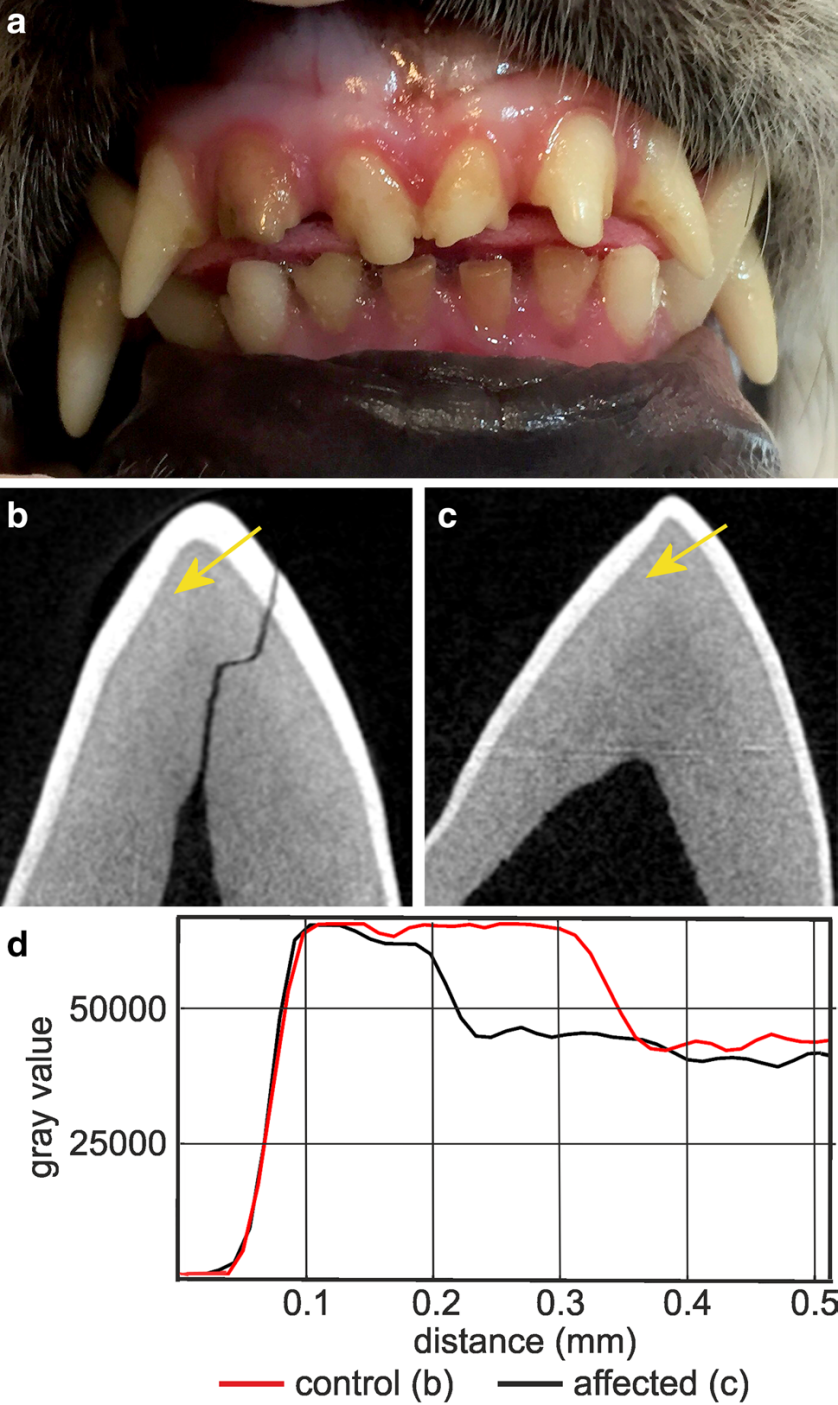
Enamel hypoplasia in Akitas. **a** A photograph illustrating hypoplastic AI with severe abrasion of incisors in a 7-month old Akita. The photograph used with permission from Eeva Länsisola. **b** MicroCT analysis of a mandibular right deciduous third premolar (DP3) of a healthy Akita. Posterior view of the major cusp. **c** MicroCT analysis of a mandibular right deciduous third premolar (DP3) of an affected Akita. Posterior view of the major cusp. The enamel layer is generally thinner than in the healthy tooth. **d** Plots of mineral density of the control and affected teeth across the lines indicated by the yellow arrows in **b, c**. On the labial side of the cusp tip the enamel thickness of the affected tooth is reduced to 50% of control. Mineralization of the enamel is slightly reduced while the dentin appears normal

**Fig. 4:**
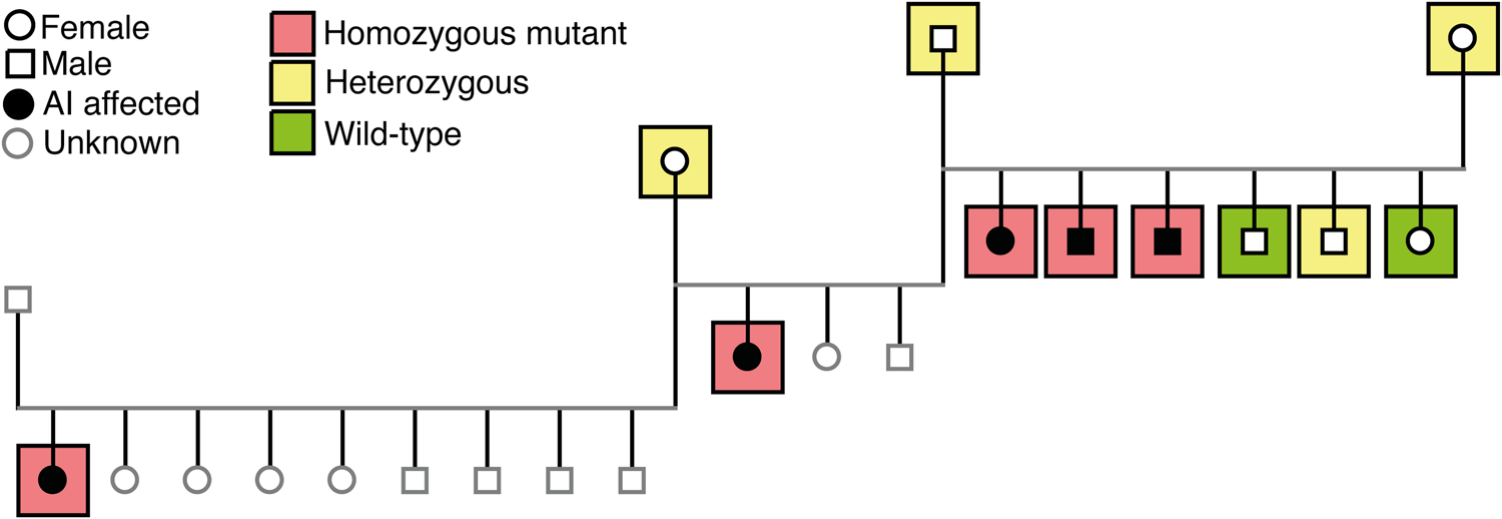
Pedigree constructed around five Akitas affected by amelogenesis imperfecta. The affected dogs are closely related and both females and males are affected. The *ACP4* variant segregated in the pedigree according to a recessively inherited disease

Six Akitas were diagnosed with AI which caused severe abrasion of the permanent teeth (Fig. 3a). MicroCT analysis of exfoliated deciduous premolars indicated variable extent of enamel layer hypoplasia as compared to a healthy control deciduous premolar (Fig. 3b–d). The dental layer appeared normally mineralized while in the enamel layer, there was slight hypomineralization (Fig. 3d). Pedigree data suggested an autosomal recessive disease (Fig. 4).

#### Homozygosity mapping and WES identified an ACP4 variant

**Fig. 5:**
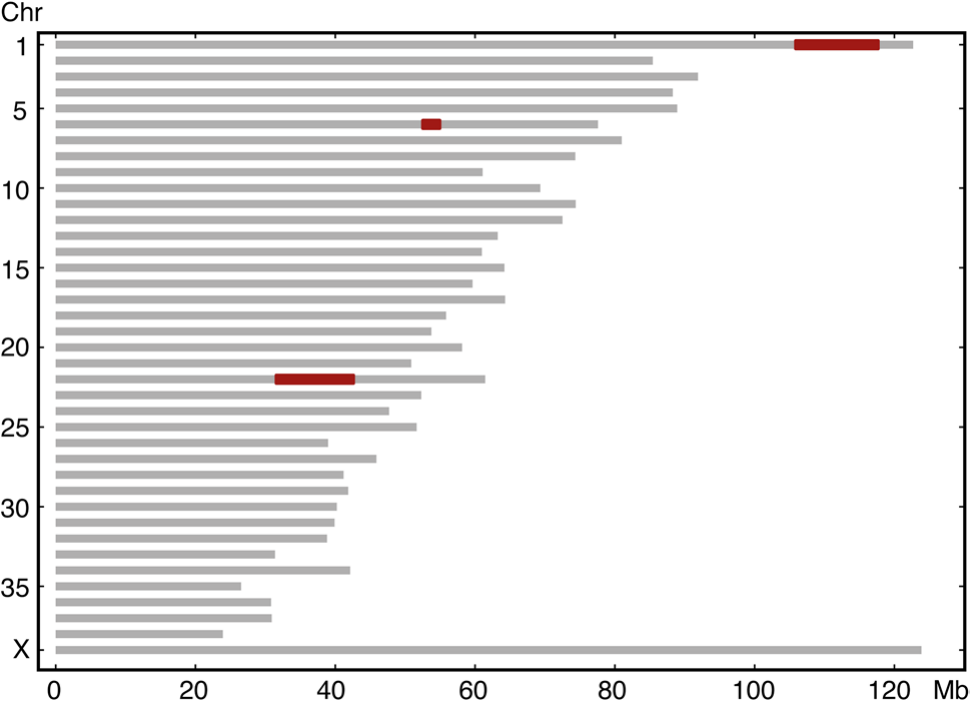
Homozygosity mapping resulted in three ROHs, in chr1 (105,875,051–117,668,302 bp), 6 (52,522,808–54,942,283 bp) and 22 (31,559,668–42,601,865 bp) (marked in red)

**Fig. 6:**
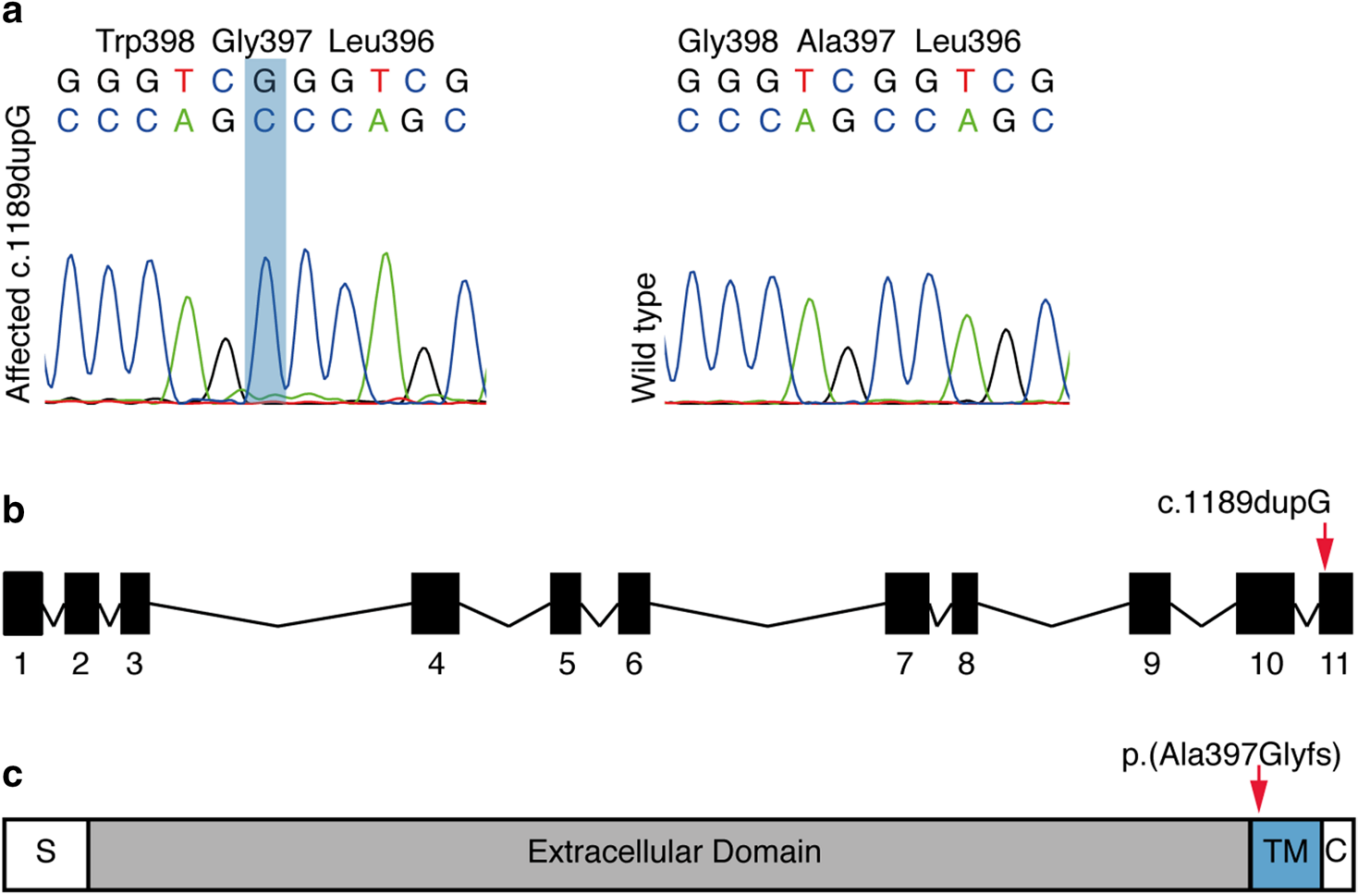
Identification of a duplication in *ACP4* associated in AI in Akitas. The Sanger sequencing chromatograms showing c.1189dupG variant as homozygous in the affected dog and wild-type in the unaffected dog. **b***ACP4* gene contains 11 exons and the variant is located in the last exon. **c** The variant is predicted to lead to a frameshift changing the amino acid sequence starting from the extracellular end of the transmembrane domain (amino acids 395–415)

To identify the genetic cause of AI in Akitas, we first genotyped five affected and eight unaffected dogs using Illumina’s HD 173K SNP array to identify runs of homozygosity (ROH). The quality control for the genotyping data was carried out and homozygosity mapping was performed on five cases. The analysis revealed only three ROHs shared by the cases (Fig. 5). We performed WES on two affected dogs and filtered the variants assuming an autosomal recessive inheritance and using 648 dog and wolf genomes from different breeds as controls (Online Resource 1). The filtering resulted in 112 variants from which 12 located on loci identified by homozygosity mapping (Online Resource 4). The 1-bp duplication in *ACP4* (c.1189dupG) was the most plausible one because of the known association of *ACP4* with AI in humans. The variant is predicted to lead to a frameshift and aberrant amino acid sequence from the aa position 397 onwards, p.(Ala397Glyfs) (Fig. 6). The variant disrupts the transmembrane domain of the ACP4 protein and, if translated, results in stop-loss according to an Ensembl and NCBI gene prediction. Because the predictions of ACP4 lack the 3′ UTR sequence, we used also AUGUSTUS gene prediction (AUGUSTUS ab initio gene predictions v3.1), which contains the 3′ UTR. This analysis proposed an altered protein with 91 extra amino acids, disrupting the C-terminus including most of the transmembrane domain.

To evaluate the segregation pattern, we genotyped the variant by Sanger sequencing in a cohort of 159 Akitas including 6 affected dogs and 153 control samples from our biobank. All affected dogs were homozygous for the variant. Among the control population, we found two dogs, littermates, that were homozygous for the variant and the rest were either heterozygous (*n* = 36) or homozygous (*n* = 115) for the wild-type allele (Fig. 4). We also screened the variant in a cohort containing samples from 78 dogs from three-related breeds, including American Akitas (*n* = 197), Alaskan Malamutes (*n* = 36), Kai (*n* = 9) and Hokkaido (*n* = 3). The screening revealed one homozygote and 44 heterozygotes in American Akitas and no variants in other breeds. The two Akitas and one American Akita that were homozygous for the variant were confirmed to be affected by AI. The carrier frequency was calculated to be 22% in both Akitas and American Akitas. In summary, the *ACP4* variant fully segregated with AI in the studied breeds.

## Discussion

Unlike rodents with continuously erupting incisors, dogs provide physiologically more comparable models of human dental physiology and AI having both deciduous and permanent teeth. Therefore, gene discovery in canine AI may provide a complementary approach for genotype–phenotype correlations and understanding of the pathophysiology. Affected dogs could also serve as potential preclinical models for currently lacking treatment scenarios. This study reveals two new canine AI variants in *ENAM* and *ACP4* genes. Both of these genes play a significant role in the secretory stage of amelogenesis and their defects lead to enamel hypoplasia. The discovery of the first spontaneous ACP4 deficiency in animals is of particular interest since the association of *ACP4* has been only recently reported in humans and no mouse model exists yet.

*ENAM* is a well-established AI gene across species (Smith et al. 2017a) and presents here a strong candidate for the recessive AI in the affected PRTs with a matching hypoplastic clinical pathology. Enamelin is a large (> 1100 amino acids) evolutionarily conserved glycoprotein expressed by secretory stage ameloblasts (Hu and Yamakoshi 2003). It is the largest of the three enamel matrix proteins, representing 1–5% of the total enamel protein within hydroxyapatite crystals (Stephanopoulos et al. 2005). Enamelin undergoes proteolytic processing to yield functional products of various sizes (186, 155, 142, 89, 34, 32 and 25 kDa) (Fukae et al. 1993; Hu and Yamakoshi 2003; Lu et al. 2008). The identified variant results in an amino acid change, p.(Pro239Leu) at a conserved residue in the first quarter of the canine ENAM protein. The missense change was predicted as highly deleterious, likely affecting the protein structure and function to cause enamel hypoplasia. However, further studies are needed to establish the pathophysiology of the p.(Pro239Leu) change. A growing body of evidence demonstrates that *ENAM* variants may not affect only the enamel matrix itself but also lead to the protein’s abnormal intracellular processing and unsuccessful unfolded protein response (Brookes et al. 2014).

Our canine AI model is recessive and fully penetrant. The affected dogs were all homozygous for the *ENAM* variant. Recessive *ENAM* models are important to better understand ENAM functions and phenotypic differences across species. *ENAM* is a major contributor in AI etiology with many reported variants, however, most of these are associated with autosomal dominant AI. Recessive AI forms have been associated with generalized hypoplasia, whereas milder localized phenotypes segregated as dominant traits (Ozdemir et al. 2005; Hart et al. 2003). The canine recessive *ENAM* model does not display such zygosity effect being fully penetrant recessive traits. The first canine *ENAM* variant described in Italian Greyhounds was a 5-bp deletion resulting in a predicted truncated protein [p.(Phe665Argfs*3)] (Gandolfi et al. 2013) with similar enamel hypoplasia described in the affected PRTs. However, there are no reports from veterinarians, breeders or owners describing milder, dominant AI traits in affected Italian Greyhounds or PRTs.

This study also discovered the first spontaneous AI animal model of *ACP4* and provides further evidence for the significance of *ACP4* in amelogenesis. *ACP4* has been recently renamed as it was previously called testicular acid phosphatase *ACPT* (MIM _606362). It encodes a 426 amino acid protein and is expressed by secretory ameloblasts and an odontoblast cell line with a suggested role in odontoblast differentiation and mineralization during dentine formation (Seymen et al. 2016; Choi et al. 2016). However, the function of ACP4 during amelogenesis is currently poorly understood. No mouse model has been characterized, but recent reports have described seven missense variants with autosomal recessive hypoplastic AI in humans (MIM #617297) (Seymen et al. 2016; Smith et al. 2017b). Our results also indicate hypoplasticity of enamel as the principal feature of enamel associated with the loss of ACP4 function. The known human variants affect residues within the extracellular domain of ACP4 while the canine frameshift variant affects the C-terminal end of ACP4, plausibly leading to loss of cell membrane inclusion of the ACP4 protein, which in turn may account for the hypoplastic enamel observed in the affected dogs.

In conclusion, we describe here two novel canine models of human AI with recessive variants in the *ENAM* and *ACP4* genes. The study supports the association of these genes with AI and provides insights to genotype–phenotype correlations. Given the comparable dental physiology between dog and human, canine models could provide functional resources for further AI research and serve as preclinical models for potential therapeutic options currently lacking in AI. Meanwhile, genetic tests devised from the findings will improve veterinary diagnostics and breeding scenarios to avoid affected dogs in future populations of the identified AI-susceptible breeds.

## Electronic supplementary material

Below is the link to the electronic supplementary material.

Canine control variant data used in the study (XLSX 11 KB)

The homozygous case-specific variants with predicted effect on protein in AI-affected PRT (XLSX 10 KB)

Multiple alignment of the *ENAM* variant region across species (TIF 992 KB)

The homozygous case-specific variants in AI-affected Akitas (XLSX 10 KB)
