# Supplementary figures and images for: Canine models of human amelogenesis imperfecta: identification of novel recessive ENAM and ACP4 variants
Source: Hum Genet. 2019 Mar 15;138(5):525–33. doi: 10.1007/s00439-019-01997-8 (PMC6536466; doi:10.1007/s00439-019-01997-8)

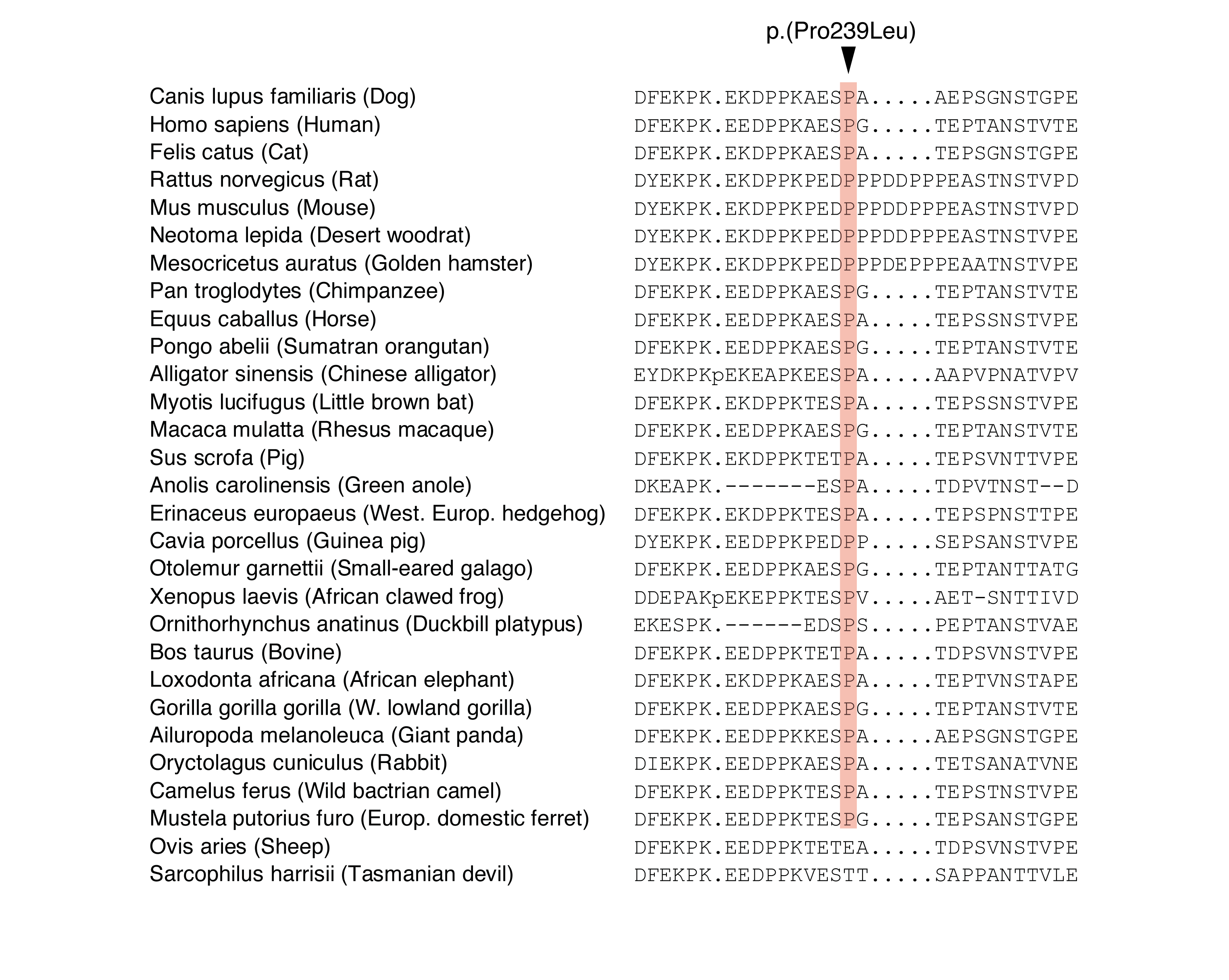

Supplement: Supplementary file 3 — Multiple alignment of the ENAM variant region across species (TIF 992 KB) [file 439_2019_1997_MOESM3_ESM.tif]
